# Prevention and Management of Complications and Education in Endoscopic Submucosal Dissection

**DOI:** 10.3390/jcm10112511

**Published:** 2021-06-06

**Authors:** Yoshitsugu Misumi, Kouichi Nonaka

**Affiliations:** Department of Digestive Endoscopy, Tokyo Women’s Medical University Hospital, 8-1, Tokyo 162-8666, Japan; yoshikft34@gmail.com

**Keywords:** endoscopic submucosal dissection, complication

## Abstract

Endoscopic submucosal dissection (ESD) is considered superior to endoscopic mucosal resection as an endoscopic resection because of its higher en bloc resection rate, but it is more difficult to perform. As ESD techniques have become more common, and the range of treatment by ESD has expanded, the number of possible complications has also increased, and endoscopists need to manage them. In this report, we will review the management of critical complications, such as hemorrhage, perforation, and stenosis, and we will also discuss educational methods for acquiring and improving ESD skills.

## 1. Introduction

Endoscopic submucosal dissection (ESD) is a form of endoscopic resection that originated in Japan as a treatment for early-stage gastric cancer [1]. Today, ESD has become an accepted treatment not only in Japan but also in other Asian countries and around the world [2]. Its treatment targets are not limited to gastric cancer but also include esophageal cancer, colorectal cancer, pharyngeal cancer, and duodenal cancer. Precancerous lesions and early stage cancers of the gastrointestinal tract that are unlikely to metastasize to the lymph nodes, regardless of the organ, are targeted for ESD. It may also be used to treat residual or recurrent disease. Although ESD is considered superior to conventional endoscopic mucosal resection (EMR) in terms of its high en bloc resection rate, it is more difficult to perform than EMR and requires more delicate endoscopic techniques [3,4]. This inevitably increases the possibility of treatment-related accidents, such as bleeding and perforation. In the case of ESD for esophageal cancer, if the circumference of the mucosal defect after treatment is larger than three fourths of the circumference of the lesion, there is a 90% probability of stenosis during the postoperative healing process [5], which is one of the serious sequelae associated with ESD. The management of these serious complications is important, but prevention is the most important factor. In this article, we review the prevention and management of ESD-related accidents.

### Search Strategy

We identified studies through a literature search of the database (PubMed), with the last search performed on 31 May 2021. The key words were “endoscopic submucosal dissection” OR “ESD” AND “complication” OR “postoperative bleeding” OR ”traction” OR “perforation” OR “stenosis” OR “training”. We restricted the studies to those written in English.

## 2. Critical Complications Associated with ESD

### 2.1. Bleeding after ESD

Postoperative hemorrhage is one of the most serious complications associated with ESD. Most postoperative hemorrhages can be treated conservatively or with endoscopic hemostasis, but some cases may progress to the point where interventional radiology or surgery is required and may even be fatal, and endoscopists must manage them appropriately. In esophageal and colorectal ESD, the incidence of bleeding after ESD is low, and there are few cases of shock [6]. In the cases of gastric and duodenal ESD, it is not uncommon for shock to occur and for the bleeding site to be filled with bloody components, making hemostasis difficult [7]. The duodenum is known to have a high incidence of postoperative bleeding due to bile exposure [8]. In addition, patients on dialysis and patients taking antithrombotic drugs are known to have an increased risk of postoperative bleeding [9]. Methods to manage bleeding include drug administration, treatment of the ulcer base, and devising a response to antithrombotic agents.

#### 2.1.1. Drugs

As maintaining an intragastric pH higher than 5.4 promotes blood coagulation and platelet aggregation [10], administration of proton pump inhibitors (PPIs) or histamine H_2_ receptor antagonists (RAs) is expected to prevent postoperative bleeding in gastric ESD, as in cases of peptic ulcer. In fact, PPIs have been shown to be more effective in reducing the risk of post-ESD bleeding than H_2_ RAs [11,12] and are therefore widely used postoperatively. Furthermore, potassium-competitive acid blockers (P-CAB), which have been introduced recently, inhibit gastric acid secretion more potently and rapidly than PPIs [13], suggesting that they may inhibit postoperative hemorrhage more effectively than PPIs [14]. It has been suggested that PPIs may be useful for prophylaxis in esophageal ESD, although not to the same extent as they are in the stomach, but no significant evidence has been obtained [15].

#### 2.1.2. Treatment of the Ulcer Base

There are many reports that suturing and covering post-ESD ulcers are useful for reducing postoperative bleeding. Suturing has been reported to reduce postoperative bleeding in gastric EMR [16], colorectal ESD [17], and duodenal ESD [18], but there has also been a report that it did not significantly reduce postoperative bleeding in colorectal ESD [13]. Thus, each facility employs it following its own guidelines. In addition to the conventional method using a hemostatic clip [19], there are also methods that use an indwelling snare [20], an overstitch (Apollo Endosurgery., Austin, TX, USA) [21], an over-the-scope clip (OTSC Ovesco Endoscopy GmbH, Tuebingen, Germany) [22], and, more recently, endoscopic hand suturing [23]. Polyglycolic acid (PGA) sheets are often used to cover the ulcer base with a special material after ESD, and they have been reported to be useful even in patients taking antithrombotic drugs [24,25]. 

#### 2.1.3. Response to Antithrombotic Agents

With the increase in the elderly population worldwide, the number of people taking oral antithrombotic agents is also increasing. While endoscopic bleeding should be avoided as much as possible, thromboembolic events, such as cerebrovascular events, are potentially lethal and require careful handling. The Japan Gastroenterological Endoscopy Society has issued guidelines on antithrombotic agents for endoscopic examination and treatment [26]. These antithrombotic agents include antiplatelet agents, such as aspirin and thienopyridine derivatives, and anticoagulants, such as warfarin, dabigatran, and heparin. The guideline categorizes endoscopic procedures into four categories—conventional endoscopy, endoscopic biopsy, low bleeding risk gastrointestinal endoscopy, and high bleeding risk gastrointestinal endoscopy—and suggests responses on the basis of the risk of thrombosis, such as myocardial infarction and cerebral infarction, and the risk of bleeding after endoscopic treatment. ESD falls under the category of high bleeding risk gastrointestinal endoscopy. In addition, an addendum on anticoagulants, including direct oral anticoagulants, has been published [27], and preoperative measures should be taken, as shown in Table 1 in the case of withdrawal of monotherapy, in Table 2 of dual therapy, and in Table 3 of triple therapy, respectively. As vascular events, such as myocardial infarction or cerebral infarction and postoperative bleeding, can occur even if the guidelines are strictly followed, it is important to obtain sufficient informed consent from patients before treatment.

### 2.2. Perforation

Perforation in esophageal ESD may lead to mediastinitis, and perforation in gastric and colorectal ESD may lead to peritonitis, which may be fatal in some cases. The incidence of perforation in esophageal, gastric, and colorectal ESD is 3.2% [28], 2.9% [29], and 4.8% [30], respectively. To avoid intraoperative perforation, it is important to obtain a good intraoperative field of view and to reliably visualize the muscularis propria. The traction method has been reported to be useful in many such cases [31]. The clip-with-line and S-O clip methods are the main methods used. 

The clip-with-line method is a simple method proposed by Oyama et al. [32]. After the flap is formed, the endoscope is removed, and a silk line or dental floss is tied to the arm of a conventional hemostatic clip outside the body. The scope is reinserted, and the clip is attached to the flap. In this step, it is necessary to avoid accidental entrapment of the muscularis propria, and the thread attached to the flap can be pulled from outside the body with a small amount of force to ensure a good field of view and reliable visualization of the muscularis propria. The proximal side of the colon is generally not a good indication for this method because of interference with the scope. Reinsertion of the scope is necessary in the original method, but a modified method that does not require reinsertion of the scope has also been reported [33]. 

**Figure 1:**
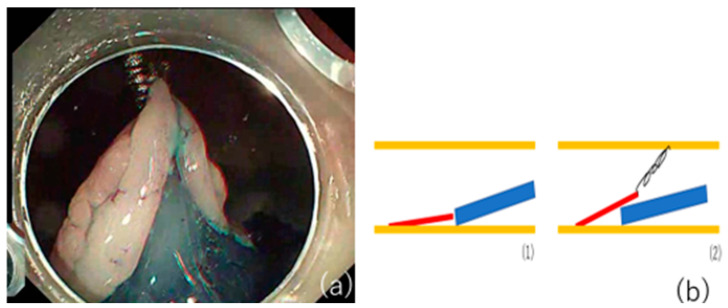
Usefullness of a S-O clip. (**a**) Good traction is obtained by using the S-O clip. (**b**) (1) Without S-O clip. It is difficult to go into the submucosal layer. (2) The submucosal layer can be easily visualized by applying the S-O clip at a site 2-fold distant from the anal side of the lesion.

**Figure 2:**
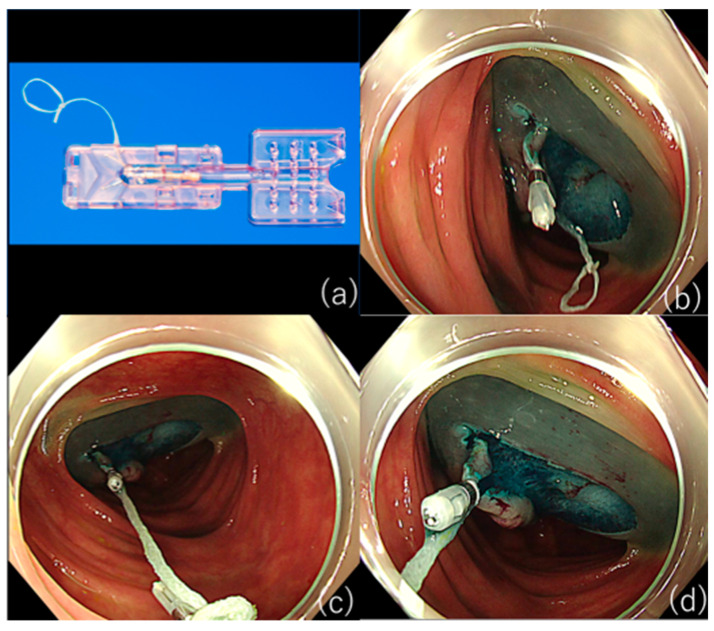
The ring-thread counter traction method. (**a**) A handmade clip with a ring made of dental floss attached to the arm. (**b**) Attach the thread ring to the flap. (**c**) Apply the ring at site 2-fold distant from the anal side of the lesion. (**d**) Good traction was obtained, as in the case of the S-O clip.

The S-O clip (Zeon Medical Co., Tokyo, Japan) is a traction device with a spring attached to the clip arm developed by Sakamoto et al. [34]. Similar to the threaded traction method, the clip arm is attached to a spring. As in the threaded traction method, the S-O clip is attached to the flap formed by mucosal incision and dissection. A small rubber ring attached to the tip of the spring is picked up with the hemostatic clip and applied to the opposite-side mucosa. Especially in the case of colorectal ESD, the rubber ring is applied to the opposite side at a site twofold distant from the anal side of the lesion to ensure a good field of view (Figure 1). The traction force can be adjusted by adjusting the air volume in the colon. After the specimen is resected, the rubber band fixed to the intestinal wall can be cut and the lesion retrieved. This method can be used in the whole digestive tract and does not require reinsertion of the scope, but the traction force may weaken as dissection proceeds [33]. However, as S-O clips are not readily available in some countries and regions, the ring-thread counter traction method, in which a loop of thread is attached to the arm of a hemostatic-like clip to obtain traction, is a relatively straightforward method (Figure 2) [35]. In both methods, care must be taken to prevent the clip from moving and accidentally grasping the intrinsic muscle layer. In the event of intraoperative perforation, care should be taken to avoid rushing to suture the perforation. If the patient’s vital signs and abdominal symptoms permit, a mucosal incision or dissection around the perforation should be made to secure space around the perforation, and appropriate closure should be performed. If there is intraoperative abdominal pain or hypotension, which may indicate abdominal compartment syndrome, abdominocentesis should also be considered. The perforation should be sutured using a hemostatic clip or an OTSC, which enables endoscopic suturing of all layers [22,36], and fasting and administration of antibiotics are necessary upon suturing of the perforation. If perforation is refractory to conservative measures, endoscopists should not hesitate to conduct surgical treatment because the perforation may be fatal.

### 2.3. Stenosis

**Figure 3:**
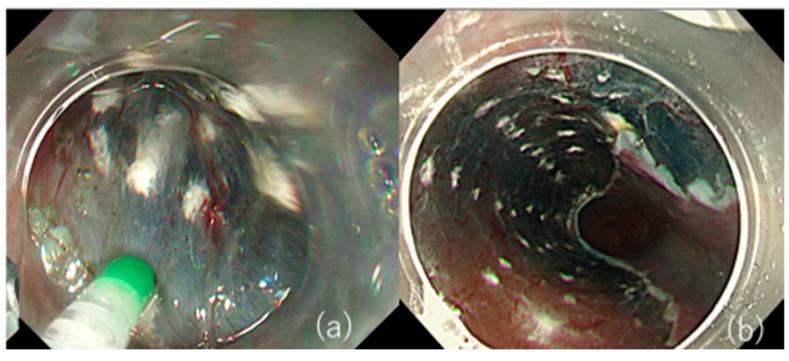
Safe local injection of TA with a spraying tube. (**a**) A spraying tube with a dull tip can be used for safe local injection by simply pushing against the submucosal loose tissue. (**b**) TA is evenly injected into the ulcer bed of esophageal ESD.

In esophageal ESD, if the circumference of the postoperative mucosal defect is larger than three fourths of the circumference of the lesion, there is a 90% probability of stenosis during the postoperative healing process [5]. Stenosis is also reported to occur in gastric [37,38] and colorectal ESD [39], although less frequently, when extensive resection is performed. Once stenosis occurs, endoscopic dilatation is frequently required, which drastically lowers the quality of life of the patient [40]. Endoscopists must therefore strive to prevent stenosis. The most important issue in clinical practice is the mechanism involved in post-ESD esophageal stricture, and the existence of a layer of horizontally oriented myofibroblasts directly above the thin intrinsic muscle layer at the site of stricture was first reported in an animal experiment [41]. Thereafter, it has been demonstrated that the same pathological change occurs in the human esophagus where post-ESD esophageal stricture develops and is later removed [42]. Myofibroblasts have been reported to have contractile properties [43], and an increase in the number of contractile myofibroblasts may be the mechanism of stricture. Hence, it is important to suppress the expression of myofibroblasts to prevent stenosis. Local administration of steroids is one of the most widely used methods of prevention. In local administration of steroids, triamcinolone acetonide (TA) is diluted to 5 to 10 mg/mL and injected into the ulcer base immediately after ESD. In general, the needle used for submucosal injection during ESD is often used, but if a needle with a sharp tip is used, TA may be mistakenly injected into the muscular layer, resulting in the risk of esophageal perforation or abscess formation [44]. Therefore, safer injection methods have been reported to avoid mistaken injection into the muscular layer, such as injecting TA with the needle retracted [45] or using a spraying tube [46] (Figure 3). Other stenosis-preventive measures include PGA sheets and cultured autologous oral mucosal epithelial sheets. PGA sheets are materials that have been safely used in the field of surgery to avoid cicatrization and adhesion [47,48]. Iizuka et al. [49] reported that PGA sheets can be applied to the ulcer base after esophageal ESD to prevent stricture formation. Furthermore, Ohki et al. [50] reported that stricture can be avoided by adjusting a sheet of ex vivo cultured autologous oral cells to the ulcer after ESD. These stenosis prevention methods should be used as appropriate, depending on what is available at each facility.

### 2.4. Education on ESD Techniques

**Figure 4:**
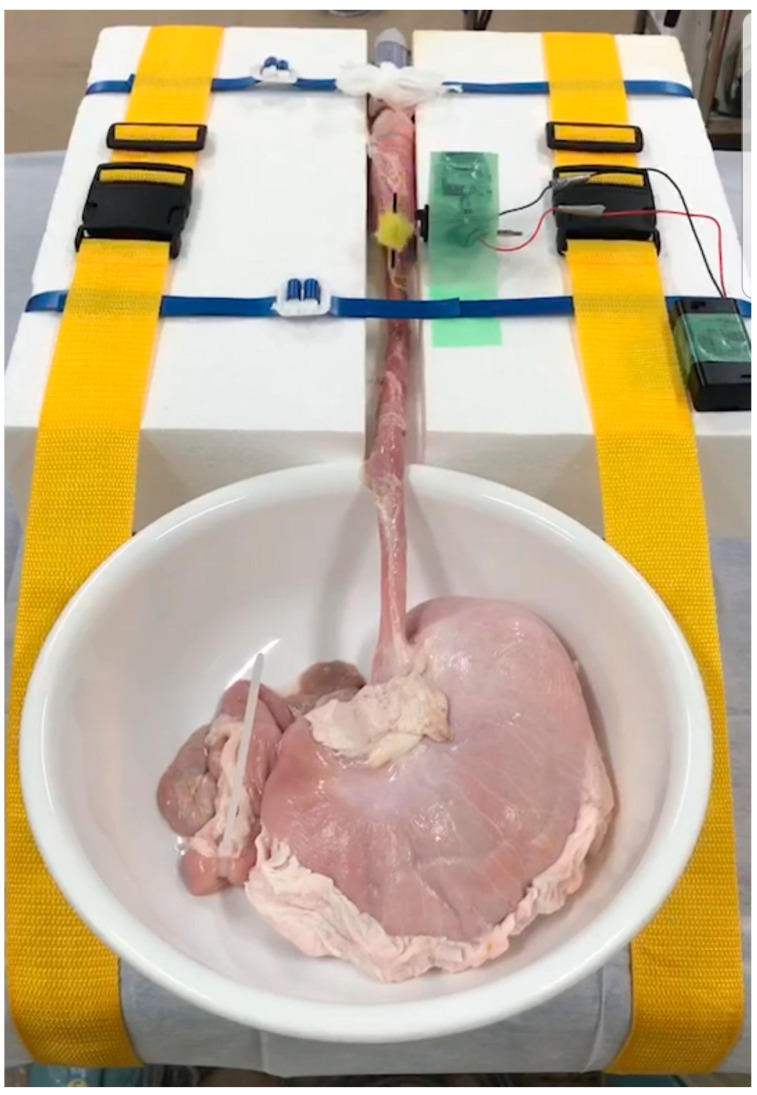
An animal model reproducing pulsations for training in esophageal endoscopic submucosal dissection.

**Figure 5:**
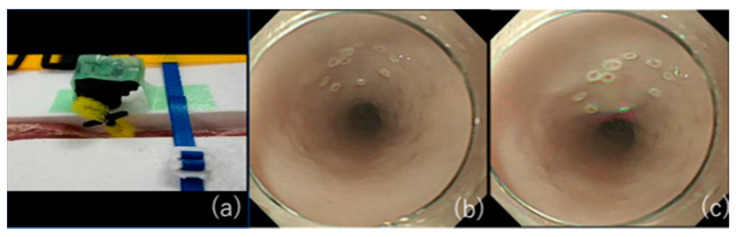
Pulsation is reproduced by the motor devise. (**a**) Pulsation is reproduced when the motor device makes contact with the esophageal wall. (**b**) Noncontact time. (**c**) Contact time. Dynamic pulsation is reproduced.

**Figure 6:**
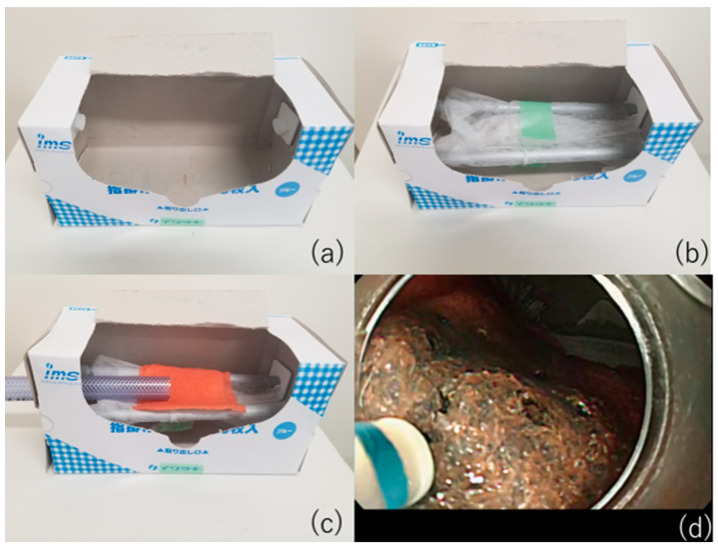
VTT ESD training model without special items. (**a**) An empty plastic apron box. (**b**) Place two rods in the box and wrap gauze around them like a stretcher. (**c**) Penetrate the hose into the box and place the VTT on the gauze. (**d**) A view during training with the VTT “konjac”, a food item model.

Although various approaches can be used to avoid accidental injury, as described above, it is fundamentally important to acquire a reliable ESD technique that does not cause accidental injury. Although it has been reported that it is generally favorable to start ESD training in the gastric antrum and the rectum because of the thickness of their walls [51], the incidence of gastric cancer is not as high in Europe and the United States as in Asia [52], and it is considered difficult to obtain a chance to improve the technique. The use of pig internal organs as a training model has been reported as a method for the acquisition of techniques before endoscopic treatment in actual clinical practice [53,54,55]. While this model is useful for acquiring basic techniques, it is certainly not realistic because of the absence of factors that make ESD difficult in actual clinical practice, such as heart rate and respiratory variability. Therefore, we have successfully developed an animal model with a heartbeat that is more in line with actual clinical practice [56] and used it to teach future clinicians (Figure 4). As the model has a motor devise that rotates at 80 cycle/min (approximately a normal human heart rate) in approximately 30 cm from the incisors of pig organ, we can perform realistic esophageal ESD training (Figure 5). It is also possible to learn about troubleshooting for intraoperative complications using ex vivo animal models. We can practice suturing a perforation intentionally made by an ESD knife with a hemostatic clip. Intraoperative bleeding, which may seem impossible to reproduce, can be reproduced by injecting red ink into the blood vessels of the organ, allowing us to practice hemostasis [57]. While some facilities and regions may have problems, such as unavailability of pig organs and cleaning of scopes used in animal models, we have also begun to conduct ESD training using Versatile Training Tissue (VTT; KOTOBUKI medical, Yashio, Saitama, Japan) made of “konjac”, a food item, which are readily available and do not require special care to clean the scope [58]. The virtual digestive tract wall is reproduced by placing VTT on a pedestal made of items found in the endoscopy lab, such as an empty plastic apron box, gauze, and rods (Figure 6). As VTT has a three-layer structure, including simulated mucosal, submucosal, and muscle layers, realistic ESD training can be performed by following the normal procedure. A training kit that includes a training model using PVA-H (EndoGel (Sunarrow Kasei Co., LTD, Tokyo, Japan)) [59] and eliminates the hygiene aspect has been reported; however, the konjac model is overwhelmingly cheaper (USD 450 vs. USD 60, respectively). Trainees need to repeat training using these in vitro models to acquire the basic ESD techniques. It is desirable to start the actual treatment from a site in which it is easy to manipulate a scope, such as the antrum or rectum. In addition, it is important for the trainee to be able to complete the ESD procedure in a small number of cases without complications by having one-on-one guidance and techniques and strategies shared with them in real time. In addition, adequate infection control measures, such as full personal protective equipment, should be taken during one-to-one guidance during the COVID-19 pandemic. 

## 3. Conclusions

Intraoperative and postoperative hemorrhage, perforation, and postoperative stenosis are critical sequelae associated with ESD, and their prevention and management were presented in detail in this review. There are multiple methods for each of these, and it is necessary to find an appropriate method for each facility. Furthermore, the establishment of training methods for ESD may be a measure to prevent complications in the long term [60].

## Figures and Tables

**Table 1:** Withdrawal of monotherapy with antiplatelet agents or anticoagulants during gastroenterological endoscopy.

	Standard Endoscopy	Biopsy	Low Risk of Bleeding	High Risk of Bleeding
Aspirin	◎	○	○	○or withdraw for 3–5 days
Thienopyridine	◎	○	○	ASA/CLZ replacement or withdraw for 5–7 days
Antiplatelet agent other than thienopyridine	◎	○	○	withdraw for 1 day
Warfarin	◎	○ therapeutic range	○ therapeutic range	○therapeutic range or heparin replacement or temporary change in DOAC
DOAC	◎	○	○	withdraw on the day of treatment or heparin replacement

ASA, aspirin; CLZ, cilostazole; DOAC, direct oral anticoagulant; ◎ = withdrawal is not required; ○ = withdrawal is required on a case-by-case basis.

**Table 2:** Withdrawal of dual therapy with antiplatelet agents or anticoagulants during gastroenterological endoscopy.

Aspirin	Thienopyridine	Antiplatelet Agent Other than Thienopyridine	Warfarin	DOAC
○or CLZ replacement	withdraw for 5–7 days			
○or CLZ replacement		withdraw for 1 day		
○or CLZ replacement			○therapeutic range or heparin replacement or temporary change in DOAC	
○or CLZ replacement				withdraw on the day of treatment
	ASA/CLZ replacement	withdraw for 1 day		
	ASA/CLZ replacement		○therapeutic range or heparin replacement or temporary change in DOAC	
	ASA/CLZ replacement			withdraw on the day of treatment
		maintain CLZ or withdraw for 1day	○therapeutic range or heparin replacement or temporary change in DOAC	
		maintain CLZ or withdraw for 1day		withdraw on the day of treatment

ASA, aspirin; CLZ, cilostazole; DOAC, direct oral anticoagulant; ○ = withdrawal is required on a case-by-case basis.

**Table 3:** Withdrawal of triple therapy with antiplatelet agents or anticoagulants during gastroenterological endoscopy.

Aspirin	Thienopyridine	Antiplatelet Agent Other than Thienopyridine	Warfarin	DOAC
○or CLZ replacement	withdraw for 5–7 days		○therapeutic range or heparin replacement or temporary change in DOAC	
○or CLZ replacement	withdraw for 5–7 days			withdraw on the day of treatment
○or CLZ replacement		Withdraw for 1 day	○therapeutic range or heparin replacement or temporary change in DOAC	
○or CLZ replacement		Withdraw for 1 day		withdraw on the day of treatment
	ASA/CLZ replacement	withdraw for 1 day	○therapeutic range or heparin replacement or temporary change in DOAC	
	ASA/CLZ replacement	withdraw for 1 day		withdraw on the day of treatment

ASA, aspirin; CLZ, cilostazole, DOAC, direct oral anticoagulant; ○ = withdrawal is required on a case-by-case basis.
